# Supplementary material for: High fidelity optogenetic control of individual prefrontal cortical pyramidal neurons in vivo
Source: F1000Res. 2012 Jul 30;1:7. [Version 1] doi: 10.12688/f1000research.1-7.v1 (PMC3894804; doi:10.12688/f1000research.1-7.v1)
Supplement: Peer review history

# Peer review: Referee response for version 1

**Published:** 2012-07-31

**Authors:** Ege Kavalali

**Affiliations:** 1 Department of Neuroscience, University of Texas Southwestern Medical Center, Dallas, TX, USA

Overall, this is a well-executed study that fits the publication objectives of *F1000Research*.

The authors report *in vivo *single unit recordings from cortical prelimbic pyramidal neurons expressing channelrhodopsin-2 or halorhodopsin. Although the basic premise of this article is not entirely novel and the authors refrain from arriving at any firm biological conclusions, the results raise some interesting issues. For instance, in Figure 1 and 2 channelrhodopsin-2 induced firing shows a rapid decrease in frequency (Figure 1b and 2c) which can elicit significant plasticity of the neurotransmitter release output of these neurons. Along the same lines in Figure 3, after 10 sec of green light exposure the firing frequency recovery shows significant rebound facilitation, which again could significantly impact neurotransmitter release output of these neurons. Therefore, in order to be able interpret the behavioral correlates of these activity patterns accurately, one would need to know the synaptic output properties of these neurons during and after optogenetic stimulation/silencing.

I have read this submission. I believe that I have an appropriate level of expertise to confirm that it is of an acceptable scientific standard.

# Peer review: Referee response for version 1

**Published:** 2012-07-31

**Authors:** Howard Eichenbaum

**Affiliations:** 1 Department of Psychology, Boston University, Boston, MA, USA

This is a useful confirmation of the optogentic control of neural activity in the rat prefrontal cortex.

The basic effectiveness and characterization of channel rhodopsin and halorhodopsin have been reported multiple times, so this study mainly extends the same findings to a new area of cortex in the anesthetized rat. The study design is straightforward, the results are well described, and the findings mainly solid. So, if novelty is a low priority and the hottest new technologies (in this case optical control) are the highest priority, I recommend publication.

The authors should provide a more comprehensive report of previous findings in other preparations and compare results, particularly with regard to the rebound effects. Also, shouldn’t they run a light only (no virus) control to show that light itself does not drive or inhibit neuronal activity?

The distinction between phasic and not is fuzzy. What exactly is phasic about the phasic cells? They should provide a quantitative characterization.

The low baseline and evoked response rates are concerning. Is this the anesthesia?

I have read this submission. I believe that I have an appropriate level of expertise to confirm that it is of an acceptable scientific standard.

# Response

**Published:** 2012-08-03

**Authors:** Donald Cooper

**Affiliations:** Department of Psychology and Neuroscience, Institute for Behavioral Genetics, University of Colorado, USA

We thank Dr. Eichenbaum for his helpful critique of our research article. One goal we had for this research article posted in the new F1000Research science publishing model was to quickly share some of our ongoing recording datasets in order to encourage collaboration with others in the field. Our goal is to ultimately publish these datasets together with more complete analysis and conclusion and other work from our lab or new collaborators. Because we intend on publishing this data with more data and more complete analysis and conclusions we kept the analysis and conclusions to a minimum so as not to violate subsequent journal publishing policies regarding prior publication.

The question of novelty:

As was pointed out this is not the first demonstration of light-evoked activation or silencing of neural activity, so in that regard we have only successfully replicated others. However, we presented several novel findings that have not yet been reported in the literature. For example, we present novel data showing that channelrhodopsin continues to function even after 2 hrs of recording of an individual virally infected neuron. We demonstrated that CaMKII expressing (light responsive) pyramidal neurons can be classified into phasic and nonphasic based on the periodicity revealed in the autocorrelation histogram of their firing pattern and this too is novel. Overall, we described a novel protocol demonstrating light activation across a range of frequencies that may be relevant for behavioral procedures. Anyone interested in the light-evoked neural response parameters for behavioral testing can be assured that under the conditions we described high fidelity light responsiveness will be maintained. Despite these novel findings, novelty is not a criteria for the F1000Research publishing model. In fact, replication is encouraged (see http://f1000research.com/about/).

Regarding control recordings using light alone:

It was suggested that we try recording from uninfected neurons to see if light alone might drive action potential firing. We have done this several times and in the article we stated the following, “Control recordings using blue and green light (light intensities of 250 mW/mm2) did not alter spontaneous firing rates in PL neurons that did not express either ChR2 or NpHR as inferred from the lack of time-locked light responses (data not shown), which suggests no unexpected off-target effects of light alone on neuronal activity.” This data will be provided in future publications along with more analysis and conclusions.

Regarding a more comprehensive discussion of related work and comparisons of rebound responses:

We certainly intend on following this advice in future journal submissions. For this research article we do not have enough data yet to make any conclusions about whether the rebound phenomenon following halorhodopsin that has been reported in vitro actually extends to the in vivo milieu. Our data is suggestive and as we stated in the article more data is needed and these experiments are ongoing.

Low baseline firing:

The baseline firing is typical for in vivo prefrontal cortical pyramidal neuron spontaneous firing under anesthetized conditions (e.g. Laviolette and Grace, 2006). The light evoked firing was done in neurons that were selected for their sparse firing rate in order to better show the ability of light-activated channelrhodopsin to initiate action potential activity. For silencing, we explicitly chose neurons with spontaneous activity so that we could observe the silencing effect of halorhodopsin.

We are excited to contribute the the new model of science publishing F1000Research has offered and we appreciate the thoughtful comments from Dr. Eichenbaum that will improve our subsequent publication. We also welcome new collaborators resulting from this research article!
